# Mid-term evaluation of Maternal and Child Nutrition Programme (MCNP II) in Kenya

**DOI:** 10.1186/s12889-022-14627-2

**Published:** 2022-11-28

**Authors:** Patrick Codjia, Edward Kutondo, Penjani Kamudoni, Judith Munga, Aneesha Ahluwalia, Indrani Sharma, Yvon de Jong, Tom Amolo, Lucy Maina-Gathigi, Victoria Mwenda, Hemant Chaudhry, Zipporah Bukania

**Affiliations:** 1 United Nations Children’s Fund, Dar Es Salaam, TZ Tanzania; 2 Kenyatta University, Nairobi, KE Kenya; 3 IQVIA (India), IN New Delhi, India; 4 IQVIA, Gouda, NED Netherlands; 5 Kenya Medical Research Institute, Nairobi, KE Kenya

**Keywords:** Nutrition, Mid-term evaluation, Evaluation, Maternal and child nutrition, UNICEF

## Abstract

**Background:**

Kenya is faced with a triple burden of malnutrition which is multi-faceted with health and socio-economic implications. Huge geographical disparities exist, especially, in the arid and semi-arid lands exacerbated by inadequate resource allocation to the nutrition sector and challenges in multi-sectoral coordination and nutrition governance. UNICEF’s Maternal and Child Nutrition Programme is a four-year (2018–2022) resilience-building, multi-sectoral program focused on pregnant and lactating women, mothers of children under five years and children under five years. The objective of the mid-term evaluation was to establish the relevance, effectiveness, efficiency, and sustainability of the programme.

**Methods:**

The field evaluation conducted between June and July 2021, adopted a concurrent mixed-methods approach, where qualitative information was gathered through 29 key informant interviews and 18 focus group discussions (6 FGDs per population group; women of reproductive age, adolescent girls and men). Quantitatively, data were obtained through desk review of secondary data from programme reports, budgets, and project outputs where descriptive analysis was undertaken using Excel software. Qualitative information was organized using Nvivo software and analyzed thematically.

**Results:**

The findings provide evidence of the relevance of the Maternal and Child Nutrition Programme II to the nutrition situation in Kenya and its alignment with the Government of Kenya and donor priorities. Most planned programme targets were achieved despite operating in a COVID-19 pandemic environment. The use of innovative approaches such as family mid-upper arm circumference, integrated management of acute malnutrition surge model, Malezi bora and Logistic Management Information Management System contributed to the realization of effective outputs and outcomes. Stringent financial management strategies contributed toward programme efficiencies; however, optimal utilization of the resources needs further strengthening. The programme adopted strategies for strengthening local capacity and promoting ownership and long-term sustainability.

**Conclusion:**

The programme is on track across the four evaluation criteria. However, a few suggestions are recommended to improve relevance, effectiveness, efficiency, and sustainability. A formal transition strategy needs to be developed in consultation with multi-stakeholder groups and implemented in phases. UNICEF Nutrition section should explore a more integrated  programming mode of delivery through joint initiatives with other agencies under the Delivery as One UN agenda, along the more gender transformative approaches with more systematic involvement of males and females in gender-based discussions.

**Supplementary Information:**

The online version contains supplementary material available at 10.1186/s12889-022-14627-2.

## Background

As much there has been a 40% reduction in stunting among children under 5 years [1], according to the Global Nutrition Report of 2021, malnutrition persists in the under-fives. Close to 150 million are stunted, 45.4 million are wasted and 38.9 million are overweight [2, 1] Despite progress showing that 27% of 194 countries are on track to meet stunting goals, the world is off track to meeting five out of six global Maternal, Infant and Young Child Nutrition (MIYCN) targets; stunting, wasting, low birth weight, anaemia, and childhood overweight [2].

Kenya is classified as a low-middle-income country (LMIC) with a population of 47.5 M (males: 23.5 M; females: 24 M) with an under-five population of about 6 M (KNBS, 2019). In 2020, Kenya was on course to meet the Sustainable Development Goals (SDGs) for stunting, wasting, underweight and exclusive breastfeeding [3]. The stunting level reduced from 35.3% in 2008–2009 to 26% in 2014, while underweight and wasting prevalence reduced from 16.1% and 7% to 11% and 4%, respectively [4]. The policy environment in Kenya is aligned to achieve the SDGs. For instance, the current Medium-Term Plan [5] has mainstreamed the SDGs. Further, mainstreaming of SDGs in performance contracting, actions plans and sub-national County Integrated Development Plans (CIDPs), 2018 -2022, positioning Kenya to better implement the SDGs. The Government prioritized the “Big 4 Agenda” focusing on Food and Nutrition Security that accelerates SDG 2. Multi-stakeholder engagement forums such as Parliamentary Caucus on SDGs and Business, Kenya Private Sector Alliance and Council of Governors for the sub-national governments through the devolved government system that oversee the implementation and progress of SDGs as the conceptualization and implementation of interventions will be targeted to the local needs and constraints such as agro-ecological, socio-economic, and political variations. Kenya’s Beyond Zero campaign, aimed at eliminating all preventable maternal and child deaths by 2023 is also a key step towards achieving SDG 3 (good health and well-being including maternal and child health). Despite this progress, Kenya is still facing the triple burden of malnutrition characterized by the coexistence of undernutrition as manifested by wasting, underweight and stunting; micronutrient deficiencies; and overweight and obesity including diet-related noncommunicable diseases.

The Cost of Hunger study in 2019 estimates Kenya lost 6.9% of its Gross Domestic Product due to undernutrition [6]. Huge geographic disparities especially in the Arid and Semi-Arid Lands (ASAL) regions exist and are associated with the inequitable allocation of resources, chronic poverty, and cyclical emergencies. As a result of these repeated crises and limited capacities to absorb shocks, children in the ASAL areas experience multiple deprivations of their rights to health, adequate food and nutrition, and safe water amongst others—and remain disadvantaged compared to the rest of Kenya. Stunting prevalence remains very high (above 30%) in West Pokot, Kitui, Kilifi, Narok, Samburu, Mandera, Uasin Gishu, Bomet and Tharaka Nithi [4, 7].

Women’s micronutrient status and dietary diversity in the ASAL areas are poorer as compared to other regions in Kenya, and this situation has had minimal changes in the last two decades [8] Since the total government budget allocation to health increased from 7% in the financial year (FY) 2017/18 to 9.2% in FY 2018/19 [9], a gradual progression towards the Abuja Declaration target of 15% for Kenya, the nutrition sector stands to benefit from the devolution.

Despite such progress in the overall health sector funding, the nutrition sector remains grossly underfunded. Counties’ expenditure on nutrition is about 0.8% of the total county budgets which is inadequate for the nutrition sector needs in the counties.

### Maternal and Child Nutrition Program MCNP II

The Maternal and Child Nutrition Program (MCNP II) is a resilience-building, multi-sectoral program focused on pregnant and lactating women, mothers of children under five years and children under five years. The first phase of the program i.e., MCNP I was introduced in the year 2014 to 2018. Based on the key learnings from the first phase, the MNCP II was launched in July 2018.

The focus of MCNP II is for UNICEF to provide technical and financial support to the most marginalized and vulnerable areas through various ministries and government departments to ensure that: 1) communities adopt healthy infant and young child feeding behaviours and practices, as well as the demand and utilize quality nutrition services; 2) communities are provided with quality integrated nutrition services; 3) the capacity of national and county governments, and other service providers are improved, and commitment strengthened, to deliver quality integrated services; and 4) government and non-government partners adopt risk-informed integrated approaches to emergency preparedness, planning, and response to humanitarian needs [10].

## Methods

This midterm evaluation was undertaken after two years of MCNP II implementation and aimed to evaluate the relevance, effectiveness, efficiency, and sustainability of MCNP II based on the Organization for Economic Cooperation – Development Assistance Committee/United Nations Evaluation Group [10] criteria. The evaluation also identified key successes and lessons learned and covered aspects of gender, human rights, and equity sensitivity of the program.

The evaluation was executed using a non-experimental concurrent mixed method approach. The quantitative data on key indicators from the programme’s result framework were collected through a desk review of programme reports and relevant documents detailed under the data collection section.

Further, The Theory of Change Additional file 1: Annex 1 was used as a guide for the logical relationships between strategies, activities, and the results chain. Before the evaluation, a comprehensive review of processes and approaches was undertaken to understand the strengths and gaps in programme implementation and complement the evaluation findings.

The [10] evaluation matrix used included key evaluation questions, sub-questions (probes), primary and secondary key indicators and data sources. 

### Sampling design

A comprehensive mapping of the relevant stakeholders was done to understand their role in the program. Following this, purposive sampling was used to identify stakeholder groups and key informants, involved in the program implementation. Key stakeholder groups included government ministries and departments (Ministry of Health (MOH), Division of Nutrition and Dietetics (DND), Ministries of Education, Livestock, Agriculture and Fisheries, Labour and Social Protection, Treasury and Planning); implementing partners, County Departments of Health, donor agencies and private sector organizations; UNICEF representatives including decision-makers involved in program planning and design and field teams including zonal officers and nutrition support officers (NSOs); and the communities in which the program was implemented. 

Multi-stage cluster sampling was used to identify counties, sub-counties, and recruitment of participants for the beneficiary field study. 

#### Stage 1: selection of counties

**Fig. 1:**
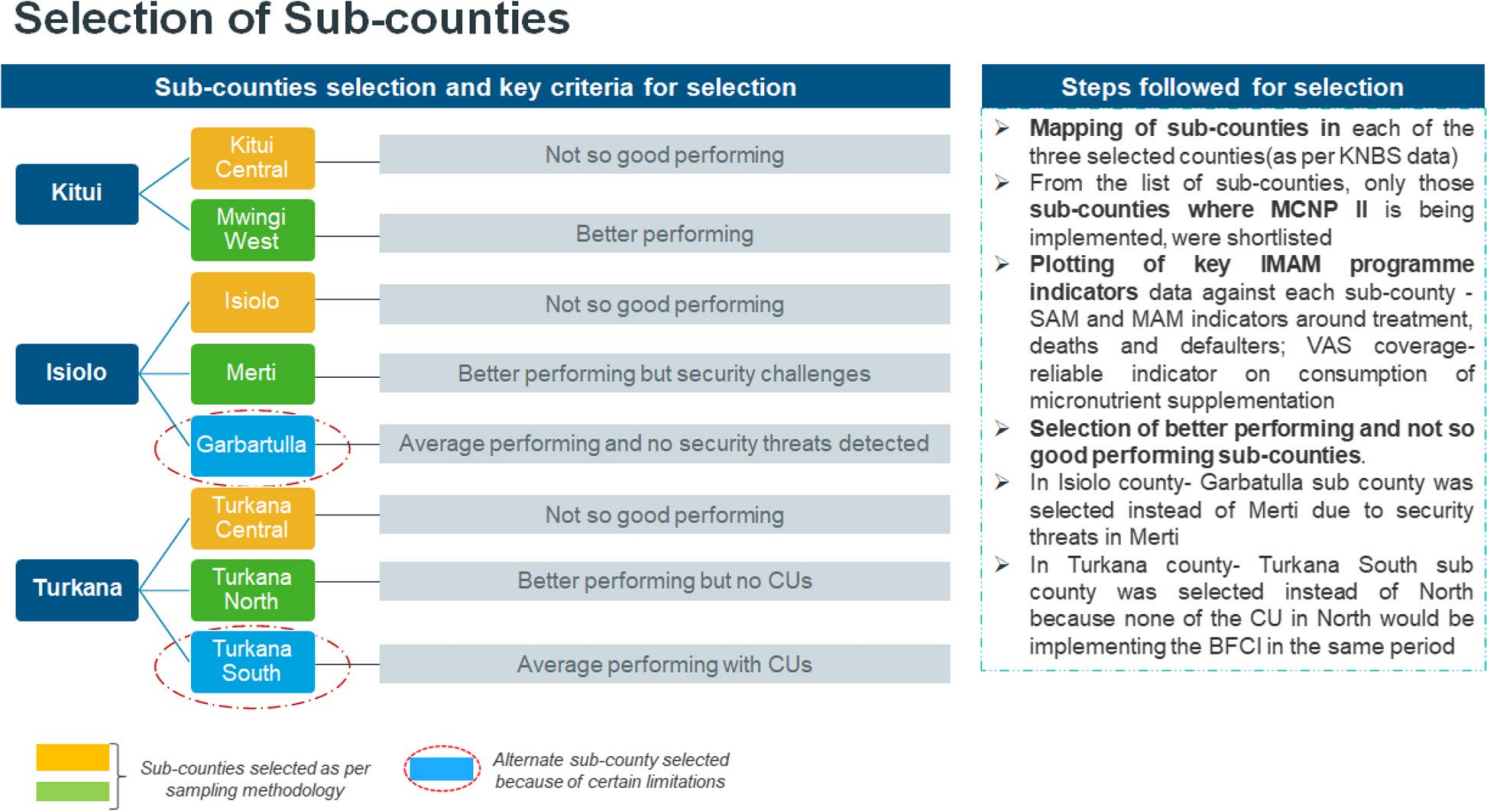
Selection of counties and sub-counties

Three counties—Kitui, Isiolo and Turkana (Fig. 1), were purposively selected from 13 program counties based on the intensity of MCNP II, levels of malnutrition, UNICEFs investment, livelihood cluster, UNICEFs field presence, partner presence, access and characteristic of the region—arid or semi-arid.

#### Stage 2: selection of sub-counties

Mapping of sub-counties in each of the three selected counties was conducted using Kenya National Bureau of Statistics (KNBS) data. The selection of sub-counties was based on the intensity of MCNP II and performance of Integrated management of acute malnutrition (IMAM) program indicators and Vitamin A supplementation (VAS) coverage. Based on these criteria, Kitui Central (Kitui), Isiolo sub-county (Isiolo) and Turkana Central (Turkana) were selected based on poor programme performance while Mwingi West (Kitui), Garbatulla (Isiolo) and Turkana South(Turkana) (Fig. 1) were selected based on better performance.

#### Stage 3: selection of participants

In each sub-county, key community groups were recruited based on their influence on nutrition and health-seeking behaviour. They included—Community health volunteers (CHVs); Community health extension workers (CHEWs); Community leaders; Mothers of children below 5 years of age; Pregnant and lactating women; Adolescent girls and Fathers/Males/Household influencers. The sample size was determined based on an assumption that saturation of information will be achieved through this sample size. The sample for qualitative design is based on the premix of saturation.

### Training and pre-testing of tools

Training the teams on data collection tools and evaluation matrix was conducted in two phases:

*Phase 1:* A two-day training for the evaluation team including qualitative researchers and note-takers to undertake key informant interviews with the stakeholder groups and *Phase 2,* one-day training for the beneficiary field study. In both phased training, a team of six were trained on evaluation tools, probing techniques, evaluation questions for key informants in the community and how to conduct focus group discussions with the beneficiaries, ethical considerations, field-level practicalities, probing techniques and note-taking. UNICEF team and members from the Expert Review Group constituted by the MoH also participated in phase 1 training as observers. the tools were pre-tested with different participants, not part of the study. In both pieces of training, debriefing sessions to discuss the flow of questions, challenges in eliciting responses and probing were undertaken. The training were conducted at the IQVIA Nairobi Office. 

### Data collection

A comprehensive desk review of key MCNP II programme documents was conducted to understand the project context, key approaches and the results achieved by the programme. The document included programme-level data sets on nutrition indicators, LMIS, nutrition action plans and budgets, MCNP II progress reports and briefs.

Through field visits, primary data using semi-structured interview/discussion guides, across two phases, to capture insights from both demand and supply sides were collected. Qualitatively, a total of 167 participants (55 males and 112 females) were interviewed. At the policy and program implementation and oversight level, the study conducted online 29 in-depth interviews through Microsoft Teams with the key informants from the selected key stakeholder groups.

At the community level, 18 face-to-face FGDs (6 per county) were conducted with beneficiaries of the program who included women of reproductive age, adolescent mothers and other decision-makers in the family (including men) at convenient levels majorly health facilities Eighteen (18). In-depth interviews with the key informants from the community including community leaders, health workers and community health volunteers (CHVs). were conducted. At the community level, tools translated into Swahili were used. Each KII lasted between 45 -60 min while the FGDs lasted between one and half hours to two hours. 

All interviews were audio recorded and notes were taken to capture insights. The interviews were sufficient, and saturation was achieved. 

COVID-19 public health guidelines were observed as provided for by SMART Interim guidance on restarting population-surveys and household level data collection in humanitarian situations during the Covid-19 pandemic” [9].

### Data analysis

All interviews were audio recorded and transcribed verbatim. Where necessary, the translations were undertaken. Stringent quality assurance mechanisms were followed to ensure the quality of data and transcripts. The qualitative data were organized, and the Inductive method was used to code and generate themes and sub-themes using NVIVO software. Quantitative data from the secondary datasets was analyzed using EXCEL. Insights were generated for comparative and trend analysis of results and program indicators. Quantitatively the review assessed the changes between the midpoint and endpoint. Analysed budget allocation versus utilization for both UNICEF funding and implementing partners’ contributions. 

## Findings

### MCNP II programme initiatives

Through key informant interviews with the programme implementers and in consultation with the MCNP II programme documents and reports, the Program activities included upstream support to key stakeholders to provide a positive enabling environment through advocacy, evidence generation, policy support, and resource leveraging. The programme supported the establishment of multi-sectoral platforms creating an environment for coordinated collaboration across the sectors of health, education, WASH, social protection, implementing partners, and the private sector. Besides governance, the programme further supported joint evidence-based planning and budgeting, service delivery monitoring and evaluation, policy change where appropriate, and emergency preparedness and response.

Through C4D, the significant role of men in balancing gender inequalities were ensured by the inclusion of men in the C4D strategies. The father-to-father support groups were formed to facilitate positive behavioural change in feeding practices and to champion the importance to seek services in health facilities. Based on positive findings of the SanNut study in the previous country programme where collaborations with the WASH sector to scale up and strengthen nutrition messaging and counselling in the Community Led Total Sanitation (CLTS) programme were scaled up under MCNP II.

The MCNP II integrated nutrition counselling into cash transfer programmes at scale to promote nutrition-centric responsive safety nets. Building upon the findings of studies conducted in the previous country programme of 2018, the programme explored and advocated for enhanced linkages with agencies focusing on agriculture and livestock regarding food production and increased access to availability and sustainability of appropriate nutritious local foods for young children throughout the year to ensure household resilience and value addition for sustained access to milk for young children throughout the year.

Community-level nutrition service delivery through the Community Health Strategy (CHS), including the Baby Friendly Community Initiative (BFCI), integrated packages of services for children and women of reproductive age, focusing on health, nutrition, WASH, and HIV services were scaled up to ensure that the pathway between improved awareness and enhanced health-seeking behaviour were not hindered by limited access to services.

### Relevance, Efficiency, effectiveness, and Sustainability (REES)

Maternal and Child Nutrition Programme II is part of the United Nations Sustainable Development Cooperation Framework (UNSDCF) 2018–2022 [11], the programme evaluation was undertaken in line with the Organization for Economic Cooperation – Development Assistance Committee/United Nations Evaluation Group [10] criteria. The findings are presented under the four evaluation criteria – relevance, effectiveness, efficiency, and sustainability (REES).

#### Relevance

Interactive discussions with stakeholders confirmed that MCNP II was relevant and aligned to the nutrition situation in Kenya; the government and UNICEF priorities, UNICEF global and regional strategies, and considered gender, equity, human and child rights perspectives.

MCNP II program design was based on a comprehensive analysis of the nutrition situation in Kenya. An analysis of the Kenya nutrition situation identified demand, supply, enabling environment and emergencies as key bottlenecks and barriers to achieving optimal nutrition for children under five and women. This informed the choice of interventions and programme focus counties for MNCP II. The ASAL counties which are prone to high levels of acute malnutrition among children under five years of age were prioritized. Further, the identified bottlenecks informed the program theory of change and alignment of program strategies to achieve the desired results.

The MCNP II result framework was found to be aligned with key Government and Ministry of Health policies including Vision 2030, Medium-Term Plan (MTP) [5], the Kenya Health Sector Strategic and Investment Plan (KHSSIP) 2014–2018 [12], Big 4 agenda [13], Food and Nutrition Security Policy (FNSP) [14] and Kenya Nutrition Action Plan (KNAP) [15]. The Programme is aligned to the social pillar of Vision 2030 on social protection, strengthening the supply chain through the Kenya Medical Supplies Agency (KEMSA) and scaling up community strategy for nutrition. Importantly the program focus on the reduction of maternal and child mortality, an objective of MTP III. While under the KHSSIP, the programme supports the objectives of reduction of mortality, the burden of malnutrition and micronutrient deficiencies, among others. Further, the program is coherent with almost all the key result areas of the KNAP.

MCNP II was aligned with the UNICEF’s Global Nutrition Strategy (2020–2030) [16] strategy that focuses on maternal and child nutrition targeting to reduce stunting [11] and builds on the UNICEF Strategic Plan, 2018–2021, and the 2016 Concluding Observations of the Committee on the Rights of the Child in Kenya. Although the program is largely aligned to these strategies, there are some areas beyond the program coverage, for instance, the burden of overnutrition and obesity; and the adoption of a lifecycle approach covering middle age childhood and elderly.

Donors reported their satisfaction as the programme’s strategic priorities were aligned to their priority focus areas such as systems strengthening and cross-sectoral integration, risk-informed programming and resilience building for nutrition emergencies as well as prioritization of ASAL counties for nutrition-specific and nutrition-sensitive programming.

All 13 target counties implemented community feedback mechanisms, including community dialogues, feedback boxes in health facilities, and other feedback processes to inform program improvements. A reconnaissance with community discussions showed that community members acknowledged the feedback mechanism and community involvement in the programme and made efforts towards gender mainstreaming, citing increased male involvement and father support groups that had been established were effective in garnering spousal support to use health services as detailed in the quotes below.

“They talk about pregnant mothers, children under five, and old age. Then as to whether such a facility is stocked with medicines or not, the community themselves sit down and get involved so they can find out. When my wife is pregnant, I take her to the clinic, she gives birth at the maternity clinic, a month later. If she is sick, I will take her to the hospital, they will bring her back to good health.” – FGD Participant (Male)

“Exactly. Mother-to-mother support. They meet to exchange ideas and support each other. At the end of the day mothers in the mother-to-mother support group are better off compared with those tucked up in the villages. Something else I want to say as a chief of this community is that this community is very vulnerable, and it is facing a lot of challenges. Despite the availability of a hospital, not everyone can get to the hospital and the available CHVs cannot manage to reach to help in every household.” – Community Leader

### Gender, equity, and human rights perspectives

Were found to be relevant aligned to the gender equality and human rights policies including session paper No 2 0f 2019 on National Policy on Gender and Development [17] under the Kenya Vision 2030 [18], Convention on Rights of the Child (CRC), [19, 20]. Convention on the elimination of all forms of discrimination against women (CEDAW) [21] as well as human rights of persons with disabilities. Key informants noted that the sex and age disaggregated data is being collected in the Standardized Monitoring and Assessment of Relief and Transitions (SMART) survey. Similarly, gender roles and maternal workload were captured through qualitative ‘Knowledge, Attitude, Behavior, Practice’ (KABP) surveys creating the opportunity for gender sensitization on the reduction of maternal workload to enhance nutrition outcomes in the communities. However, a systematic approach needs to be adopted to include women in programme design and conduct gender-based discussions [22, 23, 17–21].

### Effectiveness

Effectiveness relates to the utilization of resources to achieve the intended results. For the MCNP programme the effectiveness was identified on MCNP programme approaches used, advocacy, and value for money for cost-effectiveness. Table 1 provides a comparative analysis between the planned and achieved targets for the 13 focus ASAL counties except for admissions of children with SAM in 2020 and the proportion of facilities that offered SAM services in 2018 and 2020**.** In 2020, the sector had a SAM target expectation of 88,451 admissions, However, a lower target of 63,443 SAM admissions was achieved through MCNP II.

**Table 1:**
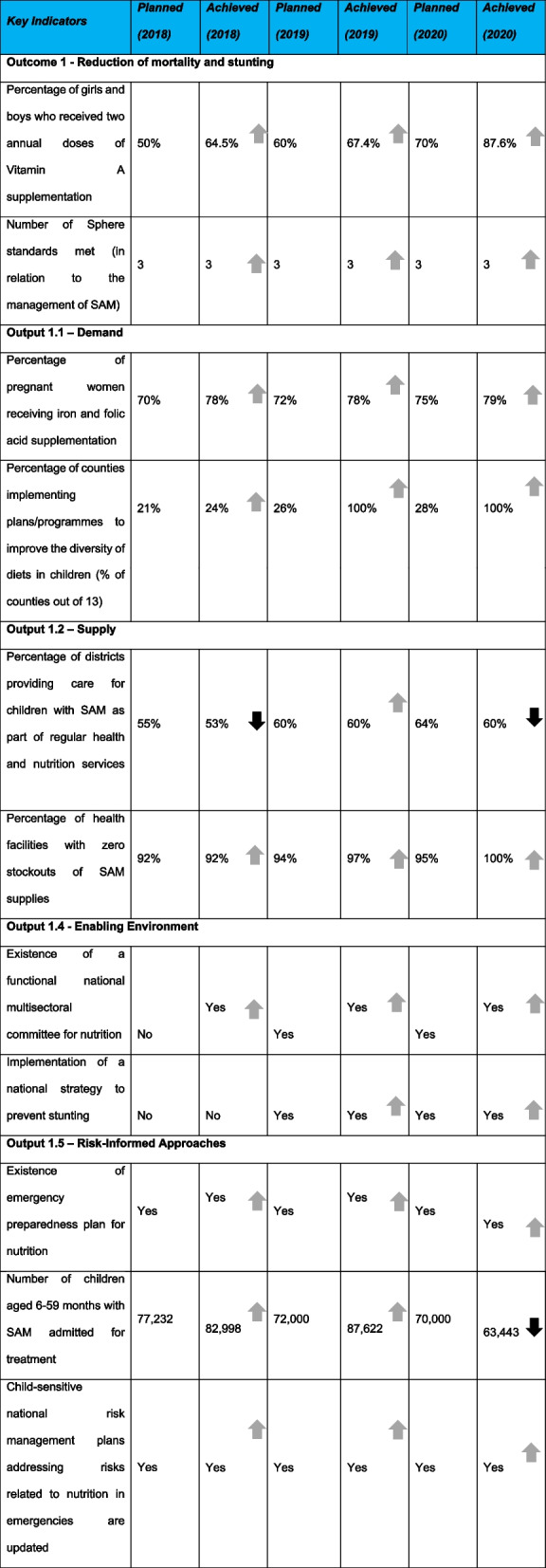
Comparative analysis between planned and achieved targets for MCNP II results

In 2018, 5 out of 13 counties were implementing plans to improve dietary diversity in children. However, in 2019 and 2020, the planned results were achieved for all 13 counties. In 2018, 7 counties had the existence of a functional national multisectoral committee for nutrition, however, this number rose to 10 counties in 2019 and in 2020, 12 out of 13 counties achieved this result. In 2019 and 2020, all 13 counties have had an existence of emergency preparedness plan for nutrition*.*

### MCNP II programme approaches and innovations

The effectiveness of the programme was associated with the service delivery approaches and innovations, use of technology and tools and alignment to government priorities as key enablers that facilitated the achievement of planned results., Malezi Bora, the child health week was used as an opportunity to reach beneficiaries for health and nutrition services including vitamin A supplementation; the family Mid-arm upper circumference (Family MUAC) for screening of malnutrition at home and self-referrals, integrated Community Management of Acute Malnutrition (ICMAM) are the service delivery models and innovations under the program. Similarly, the use of technology facilitated efficient supply chain management and improved tracking of budget expenditure. The introduction of the Logistic Management Information System (LMIS) to manage the supply chain of essential nutrition commodities contributed to achievements in zero RUTF stock-out rates in the 13 target counties; the Nutrition Financial Tracking Tool (NFTT) was critical for adequate budget allocation and tracking expenditure for nutrition sector and Rapid Pro SMS platform was leveraged for outreach and social behaviour change communication activities.

Key informants also noted that MCNP II established community peer support groups for cascading nutrition knowledge from health workers to the community. CHVs were instrumental in nutrition counselling, supporting community-facility referrals and providing support at the health facilities. Data from the focus group discussions indicated that community members perceived the provision of micronutrient supplements and nutrition counselling effective in improving service delivery. These thoughts are elaborated in the following quotes:

“…But after sensitization, the targeted mothers now know that they need to breastfeed a child for 6 months, and then introduce other foods. Also, they were not buying fruits for children, they would only give ugali with potato soup, in the morning, for lunch and dinner times. But nowadays they give fruits—the local fruits, what is available here” – HCW.

“….the community members are no longer afraid to seek medical attention, they do not fear bringing children, they have really changed” – Community Leader.

*“The community members air their problems through CHVs or Traditional Birth Attendants (TBAs); the TBA will bring their issues to the hospital and take the feedback to the community. Then the CHV will talk to the CHEW, who will talk to the In-charge. Then he will give the information to the CHEW, then disseminate it to the CHV then she takes it to the households.” – FGD Participant* [24].

### Advocacy

The programme fairly fulfilled its role to advocate for women and child nutrition rights through upstream advocacy. Advocacy led to the inclusion of more nutrition activities in county annual work plans and county integrated development plans (CIDPs) for all 13 counties. MCNP II led to the development of women and children-sensitive policies and frameworks. Advocacy efforts led to securing nutrition-specific funding in the programme-based budgets (PBB)**.** The counties of Kilifi, Wajir, Turkana, Baringo, and Samburu, now receive nutrition-specific budgets under the PBB. Further, the programme contributed to the development of terms of references (ToRs) for the multi-stakeholder platforms (MSPs), critical for cross-sectoral advocacy at the sub-national level and coordination with the national level. MSPs were functional in 12 counties, except in Kitui.

### Cost-effectiveness of implementation and value for money

Cost-minimization approaches under the programme included Training of the trainers (ToT) trainers who then cascaded the learnings to the sub-counties, reducing the cost for training all sub-county and facility level staff. On-the-Job Trainings (OJT) enabled the programme to directly reach out to the trainees (healthcare workers/facility staff) while reducing logistical costs for training. Integration of nutrition in health outreaches also emerged as a key approach to reducing costs for vertical service delivery.

To reduce the operational and overhead costs, the Value for Money (VfM) policy was leveraged under the Programme Cooperation Agreement (PCA) arrangements with the implementing partners. Before the introduction of the Value for Money (VfM) policy, implementing partners were supporting costs at about 25% of the program costs; however, with the introduction of VFM, IPs’ contribution increased. Similarly, the cost of doing business with implementing partners is reduced. Notably, UNICEF’s contribution to overhead costs was reduced, by about 12–23%. Out of the 15 implementing partners contracted from September 2018, 5 partners contributed more than 25% to the direct program costs and 10 partners contributed at least 15%, as recommended by UNICEF. To achieve value for money, implementing partner overhead ratio should be below 25%. Thus, overall, the value for money and cost-effectiveness of program implementation was moderately achieved, by the time of mid-term evaluation.

### Efficiency

Efficiency was associated with the demand and supply of nutrition commodities and supplies, stringent financial management strategies, partnership modalities and cross-sectoral integration.

Table 2 presents a comparative analysis of expenditure ratio, the amount of budget utilized in proportion to the allocated budget. The ideal expenditure ratio should be 100%. For the MCNP II, on one hand, the expenditure ratio was over 100% for the years 2018 and 2019 while on the other hand, it was about 70% for the year 2020.

**Table 2:** Budget allocation and utilization ratio across four outputs from 2018–2020

Output areas	Expenditure ratio (2018)	Expenditure ratio (2019)	Expenditure ratio (2020)
Demand	63%	17%	22%
Supply	103%	153%	0.4%
Enabling environment	135%	17%	13%
Emergencies	3%	258%	74%
**Total**	**154%**	**133%**	**71%**

The utilization of the allocated budget for demand outputs was consistently lower from 2018 to 2020, The supply outputs on the hand, the expenditure was higher than the allocated budget in the first two years of the programme (2018 and 2019) while in 2020, about 0.4% of the total budget was utilized for the supply related programme activities. This was attributed to the reduced funding following the seizure of FCDO’s support in June 2020, and the redirection of funds towards the COVID-19 response. In the first year of the programme’s inception (2018), the expenditure toward output 3 was higher than the allocated budget due to increased efforts toward creating an enabling environment. However, in both 2019 and 2020, the budget was underutilized. unlike output 3, the risk-informed programming budget was underutilized with only about 3% used in the first year of MCNP II inception. However, an improvement was noted in the subsequent years where a higher expenditure in 2019 than the allocated budget was observed. This was attributed to the programme adjustments and shifting priorities during the 2019 drought and the COVID-19 pandemic in 2020.

### Nutrition commodities and supplies

Table 3 highlights the planned v/s actual distribution of RUTF under MCNP II. The higher than planned distribution of RUTF supplies for the years 2018 and 2019, were attributed to the evolving demands and nutrition needs of the counties during the programme implementation. Situations like droughts, floods and other nutrition emergencies lead to worsening nutrition situations and increased requirements for RUTF. However, distribution was reduced in, 2020 due to COVID-19.

As shown in Table 4, in 2018, the counties of Baringo, Wajir, West Pokot, Garissa, Marsabit, Kitui, Kilifi, Kwale, Mandera, Tana River and Turkana, distributed more RUTF than the planned threshold of 100%. While in 2019 counties of Kilifi and Tana River. In 2020, Isiolo, Mandera, Wajir and Garissa, had less than 50% RUTF distributed.

**Table 3:** RUTF supplies planned v/s distributed

	**2018**		**2019**		**2020**	
**Supplies**	**Planned**	**Distributed**	**Planned**	**Distributed**	**Planned**	**Distributed**
RUTF (No)	39,063	77,529	69,814	71,811	52,474	22,707

**Table 4:**
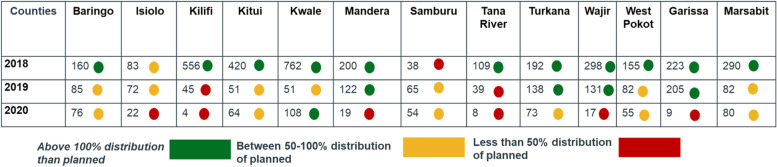
Planned v/s actual distribution of RUTF in counties (*all figures in %)

### Financial management strategies [25]

The Harmonized Approach to Cash Transfers (HACT) approach supported risk management with a focus on reducing transaction costs associated with programme implementation by harmonizing procedures as well as promoting reporting on funds that were disbursed. The funding requests were managed through Funding Authorization and Certificate of Expenditure (FACE), which required authorization from the programme managers before funding allocations. MCNP II adopted appropriate financial management procedures and approaches that collectively contributed toward bringing cost savings and efficiencies.

### Partnership modalities

The United Nations Office for Project Services (UNOPS) a partnership modality for engaging with other UN entities provided infrastructure, procurement and project management services for UNICEF to implement program activities and achieve results. Nutrition Support Officers (NSOs) were recruited through UNOPS financially and technically supported by UNICEF. NSOs were embedded in selected ASAL counties for the provision and scale-up of nutrition services, working closely with the GoK County Nutrition Coordinators (CNCs). Quantitative data revealed that engaging NSOs helped UNOPS to reduce its budget from US$4,578,433 to US$3,585,516 translating into savings of US$992,917 while achieving the same results. Importantly, the government contributed towards the programme costs through matching of funds. A total of $250 k (KES 26 M) was obtained from the Government of Kenya. The Counties of Garissa, Marsabit, Turkana, Wajir and West Pokot contributed finances for nutrition SMART surveys in 2018 and 2019. However, there is a need to enhance private sector involvement in programme planning and monitoring and evaluation**.** For instance, the partnership with the Kenya Private Sector Alliance (KEPSA) on the ‘Building Business Practices for Children’ Partnership, a tripartite partnership between the county government, Unilever and UNICEF to scale Baby Friendly Community Initiative (BFCI) models across industries is one of the key examples of private sector involvement to improve quality, coordination and efficiency.

### Cross-sectoral integration

The four thematic/result areas of demand, supply, enabling environment and risk-informed/shock responsive programming focus of MCNP II, have provided opportunities for integration and cross-sectoral programming with health, WASH, livestock and agriculture, education, child protection and social protection sectors as outlined in Table 5. The project bolstered the existing community sanitation initiative with a set of nutrition behaviour-change messages targeted at caregivers of young children. Evaluation of the project found that it improved families’ sanitation practices and nutrition knowledge [23] without adversely affecting other sanitation components, UNICEF scaled the integrated sanitation and nutrition programme to the second county in Kenya, West Pokot. In addition, implementation of the combined programme helped to reduce implementation costs and scale up at a more accelerated pace.

**Table 5:** Synergies with nutrition-sensitive sectors

Health	WASH	Livestock and Agriculture	Education	Child Protection	Social Protection
• Integrated community case management (iCCM) **•** Baby Friendly Hospital Initiative (BFCI) at basic and comprehensive emergency obstetric care facilities **•** Integration of vitamin A supplementation (VAS) into expanded immunization programme supply chain **•** Integrated management of acute malnutrition (IMAM) surge and integrated outreach **•** Integration of nurturing care in BFCI/Maternal infant young children nutrition(MIYCN)	• Nutrition integrated into community-led total sanitation (CLTS)	• Modeling and scaling of integrated programming-milk value chains food security and Agri-nutrition frameworks	• Maternal education, VAS in early childhood development centre **•** Nutrition in the school curriculum **•** Completion of education for girls	Link BFCI with birth registration	Nutrition improvements through Cash and Health Education (NICHE)

### Sustainability

As part of REES, Sustainability assessed to what extent the achievements that had been made over the first half of the programme were likely to continue even when UNICEF support for key programme areas gradually reduced. The programme review provided an insight into decentralization of processes and services, the policy environment for nutrition for children, development and integration of plans and nutrition activities at the county level and system strengthening including capacity building of national and county staff with risk programming and disaster reduction approaches.

The decentralization of processes and services to counties through the new constitution 2010, governance structure in the year 2013/2014, saw health functions devolved to the county governments. To ensure the sustainability of the provision of nutrition services at national and county levels, the MCNP II programme supported the following initiatives.

### Capacity building

Through capacity building, UNICEF trained national and county-level staff on Nutrition Financial Tracking Tool(NFTT) to address limited capacities to formulate budgets and financial plans and to improve skills in budget analysis, track expenditures and develop county budget briefs for advocacy and resource mobilization. Additionally, GOK personnel at the two levels of government were trained in the Logistic Information Management System (LMIS) for nutrition commodities to impart them with requisite knowledge and skills to forecast, request and monitor consumption of nutrition commodities at county-level. Under MCNP II, 10 out of 13 counties in ASAL regions were supported with capacity assessment and nutrition financial tracking respectively.

### Nutrition action and county integrated development plans

The programme further supported the development of plans including the National Nutrition Action Plan and County-specific Nutrition Action Plans to provide roadmaps for implementation of both nutrition-specific and sensitive interventions at the national and county-level respectively. All the ASAL counties developed county-specific nutrition action plans anchored on the national action plan. County-level leadership were engaged in the development of child-friendly legislation including Community Health Services Bills.

### System readiness assessments and strengthening

The Programme supported the development of the Nutrition Programme Maturity Analysis (NPMA) model that enabled the definition and measurement of the level of nutrition programme maturity across the 13 target counties implementing MCNPII. Assessment of system readiness using the NPMA model showed great improvements across the 13 counties between 2018 and 2020. These assessments checked counties’ readiness and self-sufficiency to gradually take up, finance and implement nutrition programmes using domestic financing. Based on the assessment, significant improvements were observed between 2018 and 2020 across each of the MCNP II counties, showing that most of the counties were on a journey to optimize programme maturity aimed at ensuring increased transition to county-led programme implementation integrated programming and cross-sectoral linkages were enhanced through support to establish or strengthen existing multisectoral technical County Nutrition Technical Forums along with the TORs in 9 out of 13 focus ASAL counties)\. However, the functionality of these forums largely depended on donor funding, hence there is a need for more domestic financing to ensure their sustainability beyond the MCNP II.

*“UNICEF has been one of our greatest supporters in terms of running the structures in nutrition, especially the convening of nutrition inter-agency coordinating meetings which are cross-sectoral. Also, the nutrition technical forum. Therefore, these are the platforms where the interventions followed by other sectors are brought to the fore. And, we have even been able to strengthen one in agriculture called food and nutrition linkage technical working group which is also now bringing together the nutrition-sensitive players in the food security and nutrition arena”-Respondent, MoH DND.*

Despite these initiatives, there are challenges such as sectoral mandates and competing priorities that hamper adequate funding allocation and implementation of nutrition interventions in the sectors. These sectoral challenges have implications for the MCNP II [23].

### Direct implementation modality

The programme engaged national and county governments to promote ownership of programme implementation and outcomes by adopting the direct implementation modality (where the Government entity as opposed to Civil Society Organizations and non-government organizations implement components of the programme directly through PCAs. The use of community peer support groups played a crucial role in strengthening and empowering community capacities through increased knowledge around nutrition and activities such as kitchen gardens for sustainability. However, the community also noted that it is important to develop community resource persons to sustain knowledge at the community level.

“…. of these groups up to now even without the support, they are continuing with support from the link facilities. So, some of the interventions are still there, they are sustainable– the mothers there are supporting one another. And the level of awareness I feel and I think though is improving. I have not done an assessment, but you know, you can tell – you are living in this community, I can say that our mothers with the different interventions which have been done geared towards nutrition, there is some level of improvement in terms of knowledge” – Respondent, CHV.

### Risk-informed programming and emergency preparedness

Notably, to ensure the sustainability of risk-informed programming and disaster risk reduction approaches, the MCNPII programme supported the development of child-sensitive bi-annual emergency preparedness response plans driven by robust information and surveillance systems at the national and county level.

MCNP II aligned its approaches and contributed to EDE in the ASALs and the Ending Drought Emergencies Country Programme Framework (EDE-CPF) pillars, particularly the Human Capital Pillar, where nutrition and health facilitated GoK’s commitment to end drought emergencies.

In addition, the programme supported the integration of essential nutrition commodities including ready-to-use therapeutic feeds (RUTF) into the GOK supply chain management system as well as scaled-up innovative approaches such as IMAM surge. Currently, 63% of the health facilities are implementing the IMAM surge model. Through MCNP II, the capacity of GoK personnel was strengthened to conduct a bi-annual food security assessment.

Under the MCNP II, UNICEF supported the MOH to develop a business continuity plan for nutrition services within the context of the COVID-19 pandemic and nutrition surveillance and information guidelines.

*“Yes, the government through the ministry of livestock, through the ministry of registration, the office of internal security usually warns us about floods, so that we can move because we will get problems.” – Community Leader.*

However, some challenges were noted that may affect the sustainability of the implementation of risk-informed programming and DRR approaches**.** These included: (1) weak multi-sectoral coordination system (2) inadequate adoption of EDE by other line ministries and stakeholders (3) inadequate mainstreaming of EDE into county integrated development plans (4) inadequate financing of innovative approaches for risk-informed programming such as IMAM surge that limited the scope and scale of coverage.

### Resource mobilization and transition strategy

UNICEF used a two-pronged approach in resource mobilization through internal and external mechanisms**.** The key donors for the MCNP II included USAID and UKAID-DFID/FCDO, EU, ECHO, and World Bank. UNICEF has over the last few years successfully implemented multi-year grants which offer flexibility in terms of programming in nutrition.

**Fig. 2:**
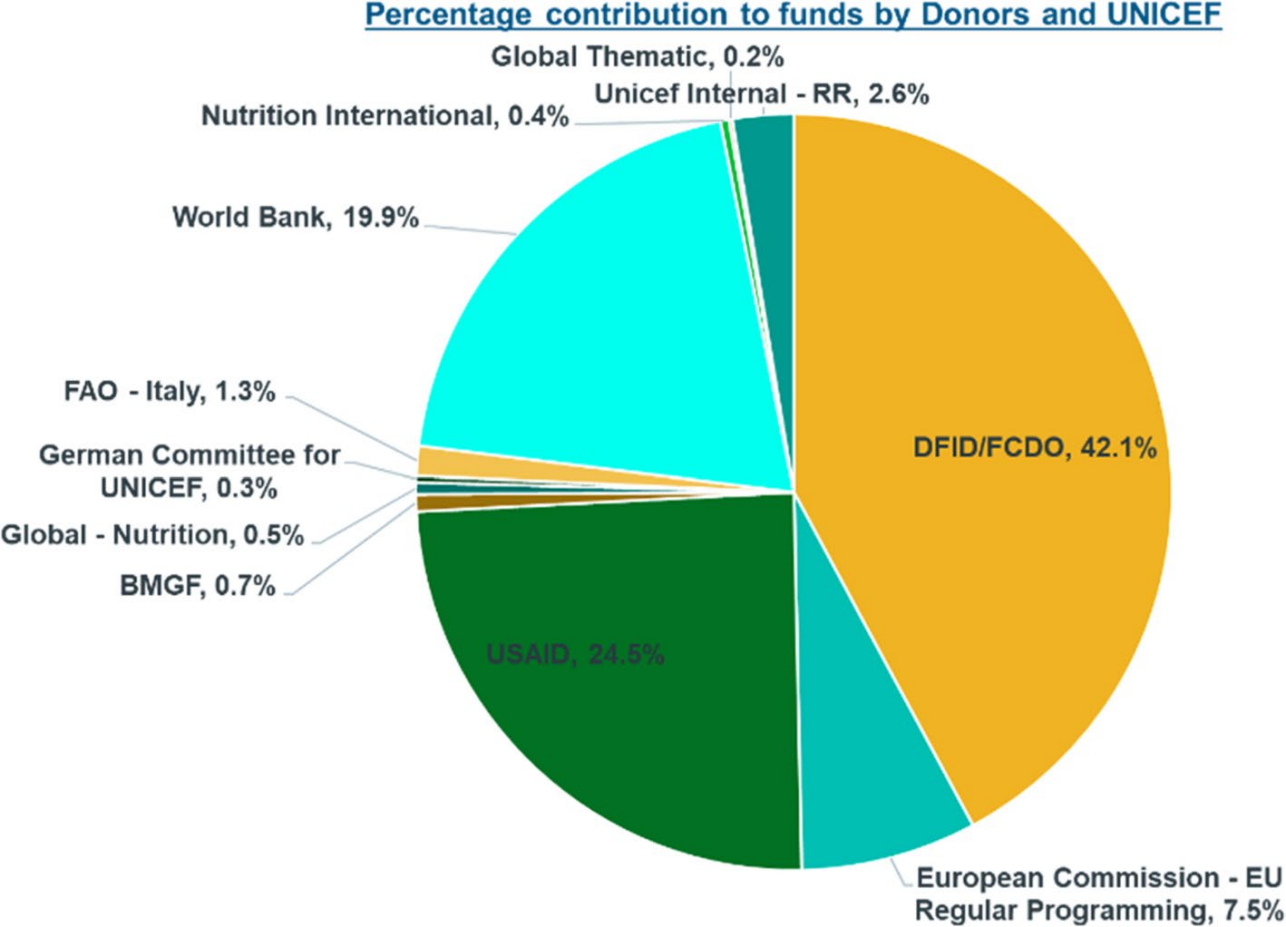
Percentage contribution to funds by donors and UNICEF *(Information Source: MCNP II database 2018–2020)*

Figure 2 gives an overview of the funding contribution by different donors and internal resource mobilization by UNICEF (2018–2020) [26].

### Lessons learned

The inclusion of males and females in nutrition programming and initiatives such as community peer support groups is gender transformative and has a positive impact on nutrition outcomes that can be scaled up and applied to other nutrition programmes.

The influence on the policy landscape and national and county resource commitment and allocation to nutrition is highly dependent on sustained advocacy for the nutrition sector priorities and needs to be adequately supported for the achievement of intended outcomes.

Cross-sectoral programming such as multisectoral Nutrition Action Plan, multisectoral coordination and multisectoral interventions such as Nutrition Improvements through Health and Education have demonstrated synergies between sectors that have proven effectiveness and efficiency in programming.

Nutrition innovations and approaches such as family MUAC, IMAM surge model, NFTT and NPMA and adaptation strategies contributed to the realization of intended results and provide learnings for other programmes.

Integrated service delivery approaches and innovations such as *Malezi Bora*, Training of Trainers and On the Job Training are cost-effective for improving service delivery and access.

Leveraging on strategic partnerships with the local CSOs and enhancing community capacity through community peer support groups, use of Community Health Volunteers (CHVs) and community feedback mechanisms were some of the other to enhance local capacity, ownership, and sustainability.

Direct implementation approaches as a health system strengthening strategy to reinforce county leadership, ownership and accountability should be emulated in programme sustainability.

## Discussion

The MCNP II program was identified as an important programme aligned with the Kenya nutrition situation and priorities and the relevant policy frameworks of the Government of Kenya. Although slightly different focus, the study by Masters et al. (2017) [27] to fill evidence gaps about the costs and impacts of nutrition-sensitive interventions relied on consultations for priority setting to ensure accuracy and relevance for policymaking, similar to the significance of MCNP II to the government [15, 11, 28, 19].

MCNP II was designed to address the geographic inequities, given its focus on the arid, semi-arid counties and urban informal settlements. Imwati and Harrison (2019) using secondary data from the 2014 Kenya Demographic and Health Survey [29] modelled various causal factors of malnutrition in ASAL areas of North Rift Kenya. The duo’s results indicated that geographical factors such as temperature, enhanced vegetation Index, Illiteracy and drinking water sources had an association with malnutrition whereas the highest association was between emperature and malnutrition. Therefore, the selection of ASAL for the MCNP II implementation where the programme objectives and plans and resource allocation were aligned to the specific county needs based on priority setting exercises was timely, thus, addressing the geographic inequities. 

The strategic approaches of MCNP II were aligned to the gender equality and human rights policies and conventions community feedback mechanisms by males and females from vulnerable communities ensured community participation. Other studies have also highlighted the central role of gender in nutrition. A study by Muraya et al. [30] stated that gender dynamics is one of the key social determinants of maternal and children’s nutrition status and is a key contributory factor to poor nutrition [31]. According to the Consultative Group on International Agricultural Research (CGIAR), gender transformative approaches orient the need to move away from burdening women with the responsibility for equality and engage men and women together as agents of change [32]. MCNP II was uniquely positioned by involving both women and men in gender-based discussions. The involvement of fathers has been shown to improve relationships with wives and fathers can become more involved in sharing responsibilities with their wives despite going against traditional norms [33].

However, the programme can be further strengthened by adopting a more gender transformative approach. Further, the programme can enhance its coherence and relevance to these strategies by expanding its scope to areas such as non-communicable diseases and adopting a lifecycle approach to include middle age childhood.

MCNP II was effective across all the four planned output areas of supply, demand, enabling environment and emergencies. By mid of the programme, the planned targets had been achieved for all the MCNP II indicators, except, the number of admissions for severe acute malnutrition in the year 2020, which was affected by the COVID-19 pandemic due to reduced hospital visits [34].

The programme moderately achieved cost-effectiveness and value for money during the first half of the programme. A systematic review by Njuguna et al. [35] found that most costs of the nutrition programs were on personnel and therapeutic feeds. The engagement of community health workers was found to be cost-effective in the treatment of uncomplicated SAM. According to Wilfold (2017) [36] and Njuguna et al. [35], the integration of outpatient and inpatient care of undernourished children through the CMAM program is cost-effective [36]. MCNP II explored integrated programming through its cross-sectoral initiatives and diversified partnerships. This is supported by a study by Levin et al. [37] and Abdulahi et al*.* [38] highlighted that multi-sectoral nutrition programmes should explore integration into routine services for economies of scale to lower costs. Similarly, MCNP II made efforts toward health systems strengthening through integrated approaches at the community level.

Despite the stringent financial cash flow management and strong monitoring strategies being adopted the optimal utilization of resources was yet to be achieved by the midterm. Resource allocation was based on priority setting exercises and comprehensive situational analysis where MCNP II adopted partnership modalities that contributed towards enhancing programme efficiencies that led to improved efficiencies and cost savings.

The gains achieved in MCNP II have been sustained across two years for all programme results. The programme has contributed to the devolution process by influencing policy, budgeting, planning and monitoring and supporting capacity development. The NSO approach at the counties was instrumental towards the achievement of results for HiNi and critical to building the capacities of the county level staff, mobilising resources, engage with leadership at the county level to advocate and direct their focus on specific areas of nutrition and support multi-sectoral coordination,

The programme provided opportunities for integrated programming with health, Water Sanitation and Hygiene (WASH), livestock and agriculture, education, child protection and social protection sectors through the scale-up of NICHE and SanNut initiatives. Prior evidence from research suggests that combined interventions for improving nutrition and sanitation practices could reduce mortality among children under five years by 15% [22, 23]. The integration helped to reduce implementation costs and scale up the combined programme at a more accelerated pace.

To strengthen systems, MCNP II focused on building local capacities and enhancing local ownership. These findings agree with other similar systems strengthening projects aimed at improving the nutrition and health of pregnant women and newborns in Kenya. According to Kung’u et al. [39], key approaches for systems strengthening include building commitment, coherence, accountability, capacity, and leadership by community sensitization and early dialogue and engagement of political and community leaders as part of stakeholder dialogue and agreement on common results' framework;

However, MCNP II lacks a formal transition strategy and hence, there is a need to develop one. While formulating the transition strategy, it will be imperative to undertake a phased approach to ensure that the process is gradual and progressive. According to FAO [40] several strategies are recommended to allow for programme transitions and ensure sustainability. There is a need to consider institutionalization of components of the programme into selected relevant sectoral activities. The nutrition activities must be included in the budgets and plans of nutrition-sensitive sectors. There is a need to assess programme resources and accordingly, plan for handover to the local governments through a consultative process including community participation to allow for institutionalization and ownership. The county government’s commitment and buy-in, especially for human resource development are crucial. Notably, Inadequate funding for emergency response due to challenges in mainstreaming the EDE has led to, delays in scaling up the IMAM surge.

Though MCNP II is made efforts to secure buy-ins from donors, there is a need to diversify partnerships. However, there is also an indication from the traditional UNICEF donors of declining support as a factor of COVID 19 impact. This calls for the need to rapidly enhance engagement with actors like the private sector to further diversify the funding basket. Similarly, there is recognition that as donor support declines, there should be a progressive increase in investment by the government. This informs the continued advocacy efforts with national and county governments to enhance public financing for nutrition.

### Limitations

The interviews with key stakeholders were conducted remotely on an online platform to minimize in-person contact as a measure to control the COVID-19 spread. Qualitative data collection on an online platform comes with limitations of rapport building with the participant and the inability to see the visual cues for probing. To mitigate these challenges, training was provided to the evaluation team including role plays for remote interviews. Underage mothers keep pregnancies in secrecy and rarely visit health facilities for fear of being ridiculed. Therefore, it was a challenge to mobilize the adolescents group for the interviews, especially where some were attending school. To mitigate this challenge, trusted community volunteers were engaged to mobilize the young mothers through their guardians. Communication with the adolescents was limited due to shyness/fear to speak despite having organized separate FGD sessions. This was mitigated through reassurance on confidentiality and the benefits of getting support. Security challenges forced a change on the selected counties replacing Merti sub-county with Garbatulla sub-county. The sub-counties were selected based on their performance on the key IMAM indicators. While Merti was a better performing sub-county than others, Garbatulla was average performing. These contextual differences in counties might have influenced the nature of the data collected and the subsequent analysis. Though the study adopted a mixed methods approach, there are certain limitations of this design such as challenges in comparison and integration of results from analysis of different data sources. Triangulation of data from multiple data sources was done to arrive at the findings.

## Conclusion

To conclude, the programme is moving in the right direction for all four evaluation criteria of REES. The findings highlighted areas that have worked well for MCNP II and have potential implications for the overall nutrition sector and other programmes. Relevance and coherence to the community and other stakeholders’ needs and the alignment to the government priorities and existing structures are key enablers for programme success. Further, gender sensitivity is critical for nutrition programming including the involvement of males and community peer support groups. MCNP II demonstrated synergies between sectors for cross-sectoral and multi-sectoral initiatives and the cost-effectiveness of Integrated service delivery approaches and innovations. The direct implementation approach worked well in terms of enhancing the county leadership, ownership and accountability. Technical and donor agencies should move towards a more enabling role and promote local capacity development for programme success. There is a need to strengthen public–private partnerships to enhance results for children. The programme has also demonstrated a successful example of public–private partnerships through the Baby Friendly Community Initiative (BFCI). However, there are larger sectoral challenges and gaps in the programme that need mitigation. As MCNP II comes to an end, there is a need to develop a transition strategy through a consultative process with all relevant stakeholders including community participation. The transition strategy can focus on areas that have worked well. In collaboration with the counties, It will be beneficial to develop resource mobilization plans that explore opportunities for multi-year funding and emerging donors while encouraging the inclusion of critical nutrition costs in the national and county budgets. There are still gaps that UNICEF can support and provide technical assistance, such as capacity development, financial planning and nutrition governance and multi-sectoral coordination mechanisms including the need to operationalize the Food and Nutrition Security (FNS) Council through sustained advocacy. Efforts should be made to achieve a more gender transformative role through systematic and sustained initiatives around gender sensitization and improving awareness that can translate into practices. There is also a need to strengthen UN guided approach of ‘Delivering as One’. The Nutrition section should  explore more inter-agency collaboration opportunities for improved nutrition outcomes and peer-to-peer learning from other UNICEF-supported programmes in Kenya.

## Supplementary Information

**Additional file 1: Annex 1.** Theory of Change.

## Data Availability

The datasets generated and analysed during the current study are not publicly available due to the nature of the data that contains audio recordings and the programme is still under implementation but is available from UNICEF on reasonable request. The request for data can be requested from the Chief of Nutrition, Kenya Office through email to co-author Lucy Maina-Gathigi at lmaina@unicef.org.

## Abbreviations

AMREF: African Medical and Research Foundation
ASAL: Arid and Semi-Arid Lands
BFCI: Baby Friendly Hospital Initiative
CLTS: Community-Led Total Sanitation
CNC: County Nutrition Coordinators
CHEW: Community Health Extension Workers
CRC: Convention on the Right of the Child
CHV: Community Health Volunteers
CSO: Civil Society Organization
CIDP: County Integrated Development Plans
CGIAR: Consultative Group on International Agricultural Research (CGIAR)
CHS: Community Health Strategy
C4D: Communication for Development
CEDEW: Convention on the elimination of all forms of discrimination against women
DND: Division of Nutrition and Dietetics
EDE: Ending Drought Emergencies
EU: European Union
ECHO: European Union, European Civil Protection and Humanitarian Operations
EDE-CPF: Ending Drought Emergencies Country Programme Framework
FAO: Food and Agriculture Organization of the United Nations
FGD: Focus Group Discussions
FACE: Funding Authorization and Certificate of Expenditure
FNSP: Food and Nutrition Security Policy
HACT: Harmonized Approach to Cash Transfers
HCW: Healthcare Worker
HINI: High Impact Nutrition Interventions
HIV: Human Immunodeficiency Virus
ICMAM: Integrated Community Management of Acute Malnutrition
IMAM: Integrated Management of Acute Malnutrition
KABP: Knowledge, Attitude, Behavior, Practice
KEMSA: Kenya Medical Supplies Agency
KEPSA: Kenya Private Sector Alliance
KHSSIP: Kenya Health Sector Strategic and Investment Plan
KNAP: Kenya Nutrition Action Plan
KNBS: Kenya National Bureau of Statistics
LMIS: Logistic Information Management System
LMIC: Low and Middle Income Countries
MCNP: Maternal Child Nutrition Programme
MIYCN: Maternal infant Young Children’s Nutrition
MOH: Ministry of Health
MSP: Multi-Stakeholder Platforms
MTP: Medium-Term Plan
MUAC: Mid-Upper Arm Circumference
NACOSTI: National Commission for Science, Technology and Innovation
NFTT: Nutrition Financial Tracking Tool
NICHE: Nutrition improvements through Cash and Health Education
NPMA: Nutrition Programme Maturity Analysis
NSO: Nutrition Support Officers
NTF: Nutrition Technical Forum
OJT: On-the-Job Trainings
PBB: Programme-based budgets
PCA: Programme Cooperation Agreement
REES: Relevance, Efficiency, effectiveness, and Sustainability
RUTF: Ready to Use Therapeutic Feeds
SanNuT: Sanitation and Nutrition programme
SMART: Standardized Monitoring and Assessment of Relief and Transitions
WASH: Water, Sanitation and Hygiene
SAM: Severe Acute Malnutrition
SDG: Sustainable Development Goals
ToT: Trainer of Trainers
TOR: Terms of Reference
TBA: Traditional Birth Attendant
UNICEF: United Nations Childrens Fund
UN: United Nations
UNOPS: United Nations Office for Project Services
USAID: United States Agency for International Development
UKAID-DFID/FCDO: Department for International Development (DFID), now Foreign, Commonwealth & Development Office (FCDO)
UNSDCF: United Nations Sustainable Development Cooperation Framework
VAS: Vitamin A Supplementation
VfM: Value for Money

## References

[1] WHO (2018). Reducing Stunting in Children: Equity considerations for achieving the Global Nutrition Targets 2025.

[2] Global Nutrition Report. 2021 Global Nutrition Report: The state of global nutrition. Bristol; 2021.

[3] Global Nutrition Report (2020). Global Nutrition Report Action on equity to end malnutrition.

[4] National Bureau of Statistics-Kenya and ICF International. 2014 Kenya Demographic and Health Survey (KDHS). Maryland, USA; 2015. www.knbs.or.ke.

[5] Government of Kenya (2018). Third Medium Trrm Plan 2018–2022 T.

[6] Government of Kenya. The Cost of Hunger: Social and Economic Effects of Child Undernutrition Kenya Country Report. NAirobi; 2019 Nov.

[7] KDHS (2014). Kenya Demographic Health Survey.

[8] Ministry of Health Kenya (2018). Kenya National Nutrition Action Plan 2018–2022 Popular version.

[9] KIPPRA (2019). Health Budget Policy Brief, No 65/2018–2019.

[10] OECD (1991). DAC Principles for Evaluation of Development Assistance.

[11] United Nations Sustainable Developement Group (2019). United Nations Sustainable Development Cooperation Framework.

[12] Government of Kenya (2014). Kenya Health Sector Strategic and Investment Plan 2014–2018 Nairobi.

[13] Government of Kenya (2020). Implementation of the Big Four Agenda Report 2018/19.

[14] Government of Kenya (2011). Kenya National Food Security Policy.

[15] Government of Kenya. Kenya-Nutrition-Action-Plan-2018–2022. 2018 Dec;1–176.

[16] UNICEF. NUTRITION, FOR EVERY CHILD UNICEF Nutrition Strategy 2020–2030. New York, USA; 2020 Dec. www.unicef.org.

[17] Government of Kenya (2019). Session Paper No 02 of 2019 on National Policy on Gender and Development.

[18] Government of Kenya (2007). Kenya Vision 2030 Popular Version.

[19] United Nations. Convention on the Rights of the Child. Geneva; 2016 Mar.

[20] UNICEF. Convention on the Rights of the Child. 1989 p. 1–15.

[21] United Nations. Convention on the Elimination of All Forms of Discrimination against Women. 1979 p. 1–10. 12289228

[22] Morrow AL, Guerrero ML, Shults J, Calva JJ, Lutter C, Bravo J (1999). Efficacy of home-based peer counselling to promote exclusive breastfeeding: a randomised controlled trial. The Lancet..

[23] Gimaiyo G, Singh S, McManus J, Lehmann L, Trevett A, Moloney G, et al. Effectiveness of integrating sanitation and nutrition (SanNut) programmes: evidence from an RCT in Kitui, Kenya. 2018;1–8.

[24] Ministry of Health Kenya (2021). The BreastMilk Substitutes (regulation and Control) (General) Regulations 2021.

[25] UNICEF. HACT-Guidance-Document-report-2018. 2018;1–64.

[26] UNICEF (2018). Communication for Development (C4D).

[27] Masters WA, Rosettie KL, Kranz S, Danaei G, Webb P, Mozaffarian D (2018). Designing programs to improve diets for maternal and child health: estimating costs and potential dietary impacts of nutrition-sensitive programs in Ethiopia, Nigeria, and India. Health Policy Plan.

[28] UNICEF. UNICEF Strategic Plan 2018–2021 Executive Summary. 2018. www.unicef.org.

[29] Mark B, Imwatis A, Harison K. Spatial Variability of Malnutrition and Predictions Based on Climate Change and Other Causal Factors: A Case Study of North Rift ASAL Counties of Kenya. J Earth Sci Clim Change. 2017;8(10).

[30] Muraya KW, Jones C, Berkley JA, Molyneux S (2017). “If it’s issues to do with nutrition. . .I. . . can decide ”: gendered decision-making in joining community-based child nutrition interventions within rural coastal Kenya. Health Policy Plan..

[31] Inés Z, Beatriz P, Clara I, Sanjay Kumar D, Sophie W, Eleanor R (2017). Gender-related barriers to service access and uptake in nutrition programmes identified during coverage assessments. World Nutrition.

[32] Wong F, Vos A, Pyburn R, Newton J (2019). Implementing Gender Transformative Approaches in Agriculture CGIAR Collaborative Platform for Gender Research.

[33] Thuita F, Mukuria A, Muhomah T, Locklear K, Grounds S, Martin SL (2021). Fathers and grandmothers experiences participating in nutrition peer dialogue groups in Vihiga County, Kenya. Matern Child Nutr..

[34] Bliss J, Lelijveld N, Briend A, Kerac M, Manary M, Mcgrath M, et al. Use of Mid-Upper Arm Circumference by Novel Community Platforms to Detect, Diagnose, and Treat Severe Acute Malnutrition in Children: A Systematic Review. Global Health: Science and Practice. 2018;6(3):1–13. Available from: www.ghspjournal.org. 10.9745/GHSP-D-18-00105 PMC6172115 30185435

[35] Njuguna RG, Berkley JA, Jemutai J (2020). Cost and cost-effectiveness analysis of treatment for child undernutrition in low- and middle-income countries: a systematic review. Wellcome Open Res.

[36] Wilford R, Golden K, Walker DG (2012). Cost-effectiveness of community-based management of acute malnutrition in Malawi. Health Policy Plan.

[37] Levin CE, Self JL, Kedera E, Wamalwa M, Hu J, Grant F (2019). What is the cost of integration? evidence from an integrated health and agriculture project to improve nutrition outcomes in Western Kenya. Health Policy Plan.

[38] Abdullahi LH, Rithaa GK, Muthomi B, Kyallo F, Ngina C, Hassan MA (2021). Best practices and opportunities for integrating nutrition specific into nutrition sensitive interventions in fragile contexts: a systematic review. BMC Nutr..

[39] Kung’U JK, Ndiaye B, Ndedda C, Mamo G, Ndiaye MB, Pendame R (2018). Design and implementation of a health systems strengthening approach to improve health and nutrition of pregnant women and newborns in Ethiopia, Kenya, Niger, and Senegal. Matern Child Nutr..

[40] FAO. Improving Nutrition Programmes An Assessment Tool for Action (Revised Edition). Rome; 2005.

