# Supplementary material for: Mid-term evaluation of Maternal and Child Nutrition Programme (MCNP II) in Kenya
Source: BMC Public Health. 2022 Nov 28;22:2191. doi: 10.1186/s12889-022-14627-2 (PMC9702643; doi:10.1186/s12889-022-14627-2)

Mid-term Evaluation of Maternal and Child Nutrition Programme (MCNP II) in Kenya

Annexes

Annex 1: Theory of Change

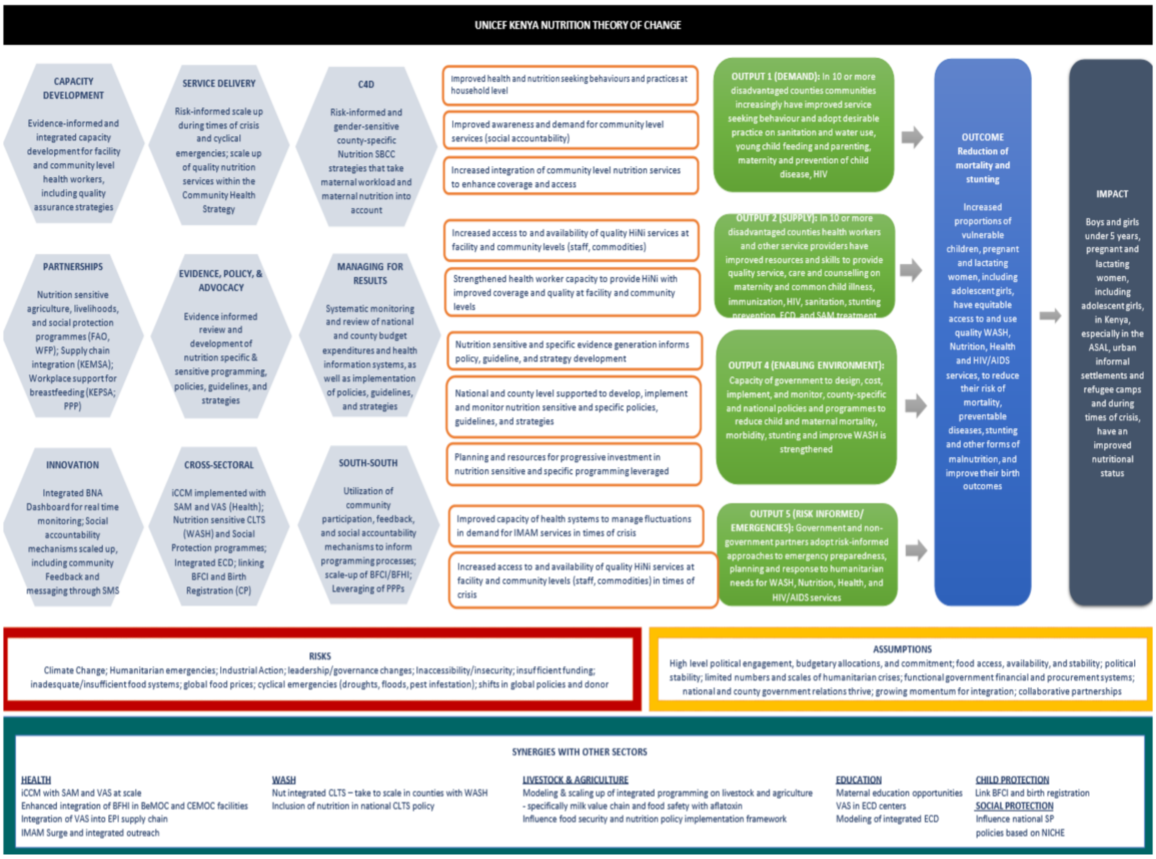

Supplement: Supplementary file 1 — Additional file 1: Annex 1. Theory of Change. [file 12889_2022_14627_MOESM1_ESM.pdf]
